# Breathing

**Published:** 1869-02

**Authors:** 


### BREATHING.

The atmosphere about us is called "Air," *the* Air, by way of distinction from other "airs," as the Sacred Scriptures, by way of pre-eminence, are called The Bible, while any other book is a bible, and any other "gas" is an air. We might with propriety and with greater perspicuity say, "Atmospheric Gas," Hydrogen Gas, Carbonic Acid Gas, Mephitic Gas. Substitute "Air" for *Gas*, and the meaning is the same.

If a man were introduced into a room filled with carbonic acid gas, he would die in a moment, as instantly as if a pillow were pressed over his face. In both cases death results from suffocation; in the latter, because he had no air at all; in the former, because the air he did breathe had no substance, no life, no nutrition in it.

The fumes of burning charcoal fill a room with carbonic acid gas in a short time, and the occupant soon dies. It is not generally known that a man would as certainly die before morning if he went to sleep in a small close room where a number of candles, lamps, or gas lights were burning. A single candle forms eight hundred cubic inches of carbonic acid gas in an hour; an averaged size man does the same in half an hour. If a man were placed in a room which measured twelve feet long, as much broad, and as much high, he would convert the whole atmosphere of it into carbonic acid gas in twelve hours if no fresh air were admitted, and he could live that long. The only reason why thousands of persons who go to bed in good health at night, in a small room, do not die before morning, is because purer air forces itself into the room at the door and window-cracks and drives it upwards through the fire place, or through any crevices that may happen to be near the floor of the room. Hence the practical necessity of having apertures in appropriate parts of all public buildings and conveyances.

"*The air feels close*," is a familiar expression. It is because it contains but little oxygen, which has either been consumed, or replaced by some innutricious substitute. The pure air may be replaced by delicious odors and perfumes, still that air is that much less nutricious; a man might soon die on a bed of roses. But to make this article practically useful to each reader, it may be suggested, and is probably true, that the person who is most susceptible to the feeling of "closeness" in entering a crowded apartment, is most liable to consumptive disease, because it shows that the lungs are working so imperfectly, that even a slight want of the natural quantity of nutriment in any breathful of air is instinctively felt; that the person is living on a minimum supply of pure air; that the amount of that supply cannot be diminished without causing instantaneous oppression to the system; and as that supply is furnished through the lungs, the reason of its not being a full supply is, that there are not lungs enough in operation to take it in. And as the usual approach of consumption is marked by the lungs working imperfectly for months and months before its actual seizure of the system, a great sensibility to the "closeness" of any atmosphere, or to the presence of odors and perfumes, should lead to the immediate adoption of daily horseback riding, running, jumping, swimming, rowing, or such other forms of pleasurable or profitable out-door activities, as are calculated to develop the fuller and freer action of the breathing apparatus. The young lady who feels so "oppressed" the moment she enters a public vehicle, that she instinctively puts out her hand to open the window, or who soonest begins to feel faint, or sick, or dizzy in a crowded apartment has, if not compressed in her clothing, good reason to apprehend that she is on the road to Consumption; while persons of mature age, whose susceptibilities are very keen in this direction, have good reason to fear that health is giving way in some part of the system. And to all we say, by all means endeavor to secure as a sleeping apartment the largest, highest, lightest, and most airy room in the house. Ventilation, light, dryness, being the indispensable requisities for a healthy sleeping-place. It is in our chambers we spend three fourths of our existence, it is in our sleep that we are most helpless against danger and most liable to the inroads of disease, for Pestilence walks in the night time, and it is in the night that most of us die. Let all then have a wise care as to their sleeping apartments, and aim, in addition, to have their sitting rooms abundantly ventilated, and besides all this, to spend two or three hours in the open air, daily.

